# Preoperative gap phenotypes in functionally aligned robotic total knee arthroplasty: derivation and internal coherence of a driver-based classification

**DOI:** 10.1007/s11701-026-03655-4

**Published:** 2026-07-20

**Authors:** Eduardo Frois Temponi, Jared Philip Sachs, Matheus Braga Jacques Gonçalves, Luiz Fernando Machado Soares, Lúcio Honório de Carvalho Júnior

**Affiliations:** 1 Hospital Madre Teresa, Av. Raja Gabaglia 1002Gutierrez, Belo Horizonte, 30441-070 MG Brazil; 2 Department of Surgical Technique, Pontifícia Universidade Católica de Minas Gerais (PUC Minas), Belo Horizonte, Brazil; 3 Department of Orthopaedic Surgery, Westchester Medical Center, New York, Valhalla USA

**Keywords:** Total knee arthroplasty, Robotic-assisted surgery, Functional alignment, Gap balancing, Knee phenotype, Classification

## Abstract

Robotic total knee arthroplasty (TKA) enables repeated intraoperative quantification of preoperative alignment, flexion deformity, and medial and lateral compartment gaps in extension and at 90° flexion before component positioning, but the preoperative patterns guiding functional-alignment planning remain incompletely characterized. This study aimed to derive a pragmatic driver-based classification from preoperative robotic data and evaluate its internal coherence across the surgical workflow. We analyzed 68 consecutive primary functionally aligned robotic TKAs performed by a single surgeon using a CT-based robotic-arm platform. Preoperative variables included hip-knee-ankle angle (HKA), flexion deformity, and independent medial and lateral gaps in extension and at 90° flexion. A 10-case formative series informed concept generation; the finalized hierarchy was then applied to all cases using preoperative variables only. Four phenotypes were derived: bone-driven, ligament-driven, flexion-dominant, and mixed/complex (defined as the co-occurrence of two or more severe deformity drivers). Internal coherence was assessed through release escalation, corrected-state behavior, plan-to-final HKA fidelity, and mechanical and functional-alignment balance. Inter-observer reliability was tested by having five independent surgeons classify all cases blinded, with agreement quantified by Fleiss’ and Cohen’s kappa. All knees were classified: bone-driven, 40/68 (58.8%); ligament-driven, 6/68 (8.8%); flexion-dominant, 10/68 (14.7%); and mixed/complex, 12/68 (17.6%). Release escalation occurred in 1/68 (1.5%), with no posterior capsulotomy. Overall, 58/63 (92.1%) finished within ± 2° of planned HKA; strict mechanical balance was achieved in 58/62 (93.5%) and functional-alignment balance in 61/62 (98.4%). Flexion-dominant knees showed lower coronal fidelity but complete final balance, suggesting a coronal-sagittal trade-off during functional planning. Inter-observer agreement among the five surgeons was almost perfect (Fleiss’ kappa 0.88, 95% CI 0.81–0.93; 92.4% overall agreement). Preoperative HKA, sagittal deformity, and medial-lateral gap asymmetry revealed recurring patterns organized into a four-phenotype driver framework. This classification is best interpreted as an internally coherent derivation framework that showed almost perfect inter-observer reliability (Fleiss’ kappa 0.88) but still requires external, multicenter validation and threshold-sensitivity analysis before its clinical utility can be established.

**Supplementary Information:**

The online version contains supplementary material available at 10.1007/s11701-026-03655-4.

## Introduction

Robotic TKA has transformed intraoperative planning from a largely experience-based process into a quantifiable workflow [1, 2] in which the native knee can be registered, tensioned, virtually planned, and assessed following trial reduction. This provides a richer dataset than conventional instrumentation or postoperative radiography alone, allowing surgeons to observe how the arthritic knee behaves before component position is optimized. This emphasis on precision is clinically motivated: despite the overall success of TKA, a substantial minority of patients—historically reported in the range of approximately 15–20%—remain dissatisfied or report unmet functional expectations after surgery, and malalignment together with residual soft-tissue imbalance are among the recognized contributors to early failure and revision. Against this background, robotic assistance has been shown in recent meta-analyses of randomized trials to significantly reduce mechanical-alignment outliers and deviation from the intended axis compared with conventional jig-based instrumentation [3], strengthening the rationale for characterizing the deformity precisely at the console before the surgical plan is finalized.

Functional alignment leverages these quantitative inputs to individualize component positioning within predefined safe boundaries while pursuing a balanced flexion-extension envelope [4, 5]. Nevertheless, an important practical question remains unresolved: before robotic optimization begins, how should the native knee be interpreted? The same coronal hip-knee-ankle (HKA) angle may represent distinct surgical challenges, including osseous deformity, ligamentous asymmetry, sagittal contracture, or a combination of severe drivers.

Existing morphological classifications are valuable for describing limb alignment and joint-line orientation [6]; however, morphology alone does not necessarily identify the primary intraoperative driver of deformity. Likewise, predictive gap-balancing and artificial intelligence-based tools may assist in optimizing a surgical plan once the problem has been defined [7], but they do not fully address how the native knee should be categorized before planning begins.

The present study was designed to make this upstream decision-making process explicit. Importantly, the proposed classification is not presented as a pre-existing taxonomy validated by the study cohort. Rather, it was developed as a derivation framework: the first 10 cases were used to identify recurring patterns and refine face validity, after which the operational hierarchy was finalized. The subsequently locked 68-case dataset was then analyzed using preoperative variables only.

The primary objective of this study was to derive a pragmatic preoperative, driver-based classification of robotic TKA cases and to evaluate its internal coherence across key downstream workflow endpoints. We hypothesized that native HKA, flexion deformity, and medial-lateral gap asymmetry would identify recurring phenotypes demonstrating distinct patterns of release escalation, corrected-state residual deformity, plan-to-final fidelity, and final gap balance.

## Methods

### Study design and cohort

This prospective, single-surgeon, single-institution cohort study included consecutive primary robotic TKAs performed with a functional alignment approach. The analysis included 68 consecutive cases with available intraoperative data capture. Reporting was conducted in accordance with STROBE guidelines for observational studies. Institutional review board approval was obtained, and all patients provided consent in accordance with local requirements.

### Robotic platform and intraoperative states

All procedures were performed using the MAKO Robotic Arm-Assisted Surgery system (Stryker, Mahwah, NJ, USA) and the Triathlon implant system [8]. Four intraoperative states were recorded. The Preoperative state was captured after registration and after removal of peripheral osteophytes (performed routinely before registration verification in all cases, as osteophytes can otherwise bias probe-based surface mapping), but before definitive component planning, formal balancing, or advanced soft-tissue release. The Corrected state recorded HKA after the standardized soft-tissue protocol and robotic tensioning. The Planned state represented the finalized virtual component plan. The Final state was recorded after bone cuts and trial polyethylene insertion.

### Sign conventions and gap variables

Varus HKA was coded as positive and valgus HKA as negative. Flexion contracture was coded as positive and recurvatum as negative. Gap values were coded as positive for laxity and negative for tightness [9, 10]. Medial-lateral asymmetry was calculated as medial minus lateral; therefore, negative values indicated relative lateral laxity. Corrected-state analyses were limited to coronal alignment because the platform displays corrected HKA but does not report corrected-state individual medial and lateral gaps in the same format.

### Soft-tissue protocol

In varus knees, a standardized partial deep medial collateral ligament (MCL) release was performed before corrected-state tensioning. This consisted of a sequential subperiosteal elevation of the deep MCL fibers from the proximal tibia, beginning posteromedially and progressing anteriorly only as needed, performed after the tibial cut and re-assessed with the robotic gap-measurement display after each increment. The release was considered complete once medial and lateral extension gaps were within approximately 2 mm of each other on the robotic display; this standardized step was always performed in varus knees regardless of severity and was not, by itself, considered release escalation [11]. Valgus and neutral knees did not undergo a routine pre-cut release [12], reflecting the more variable and less predictable lateral soft-tissue response to release in valgus deformity. Additional soft-tissue release beyond this standardized strategy — for example, a posteromedial corner or posterior cruciate ligament recession in a varus knee, or a pie-crusting of the iliotibial band or lateral collateral ligament in a valgus knee — undertaken when the robotic display showed persistent asymmetry (> 3 mm) or residual coronal malalignment (> 3°) after the standardized step and after confirming component position and resection depth were already optimized, was classified as release escalation. No posterior capsulotomies were recorded in the dataset.

### Derivation of the classification

**Fig. 1:**
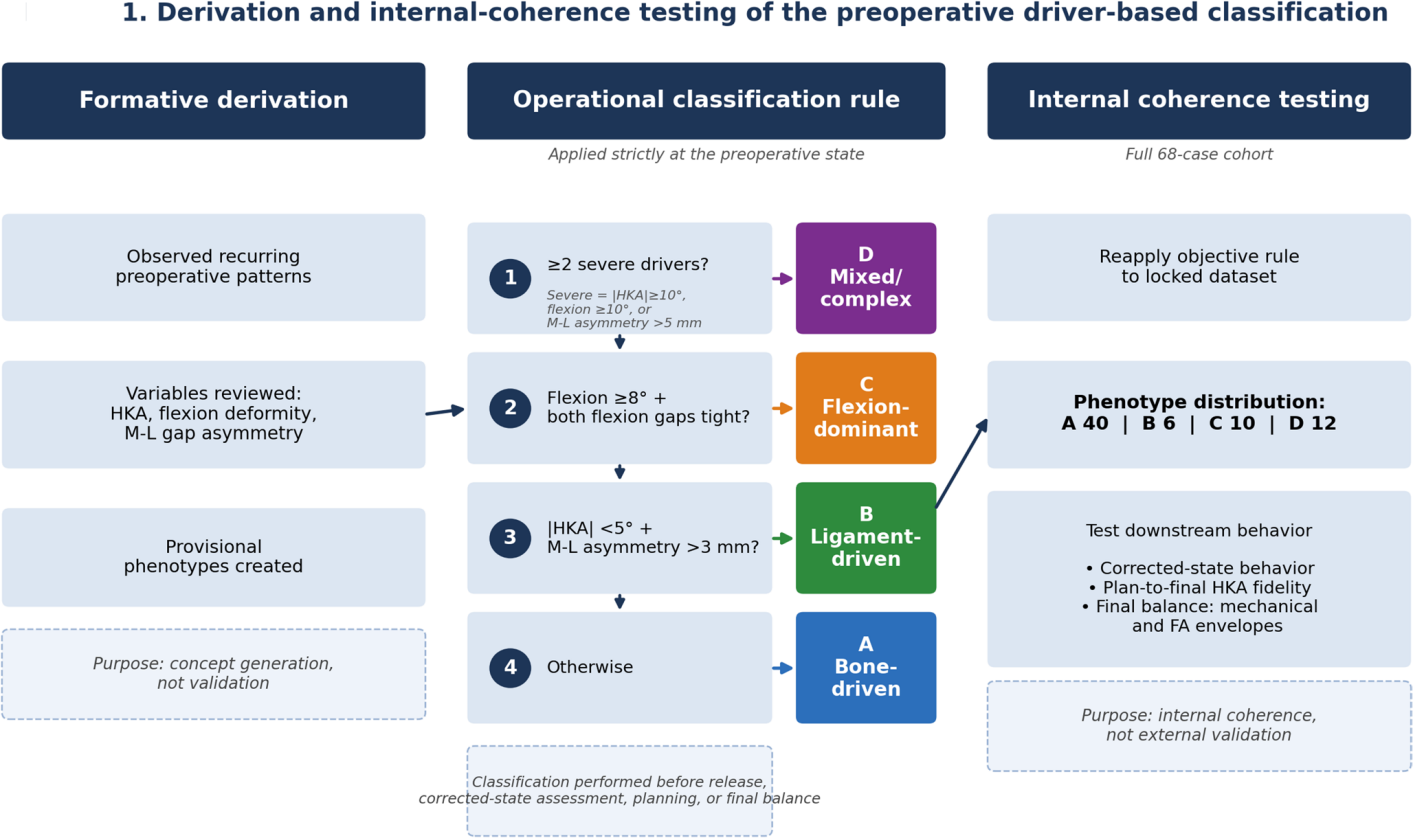
Derivation and internal coherence framework. The initial formative cases were used for concept generation and face-validity refinement. The operational hierarchy was then applied to the full 68-case derivation cohort using preoperative variables only; downstream endpoints were reserved for internal coherence checks

The classification was developed in three stages. First, an initial 10-case exploratory series was reviewed to identify recurring preoperative patterns and to transition from descriptive morphology labels to root-driver categories. In this exploratory cohort, retrospective driver assignment yielded A = 5, B = 1, C = 1, and D = 3. These cases were used for concept-generation and refinement of face-validity rather than for external validation of the classification. Second, the four driver categories were defined clinically as bone-driven, ligament-driven, flexion-dominant, and mixed/complex. Third, these categories were translated into an auditable hierarchical framework using only preoperative HKA, flexion deformity, and medial-lateral gap asymmetry (Fig. 1).

The role of the exploratory cohort was intentionally limited. The sample was sufficient to identify recurring patterns and refine classification terminology, but not to establish prevalence precision or validation of the proposed framework.

### Operational hierarchy

**Fig. 2:**
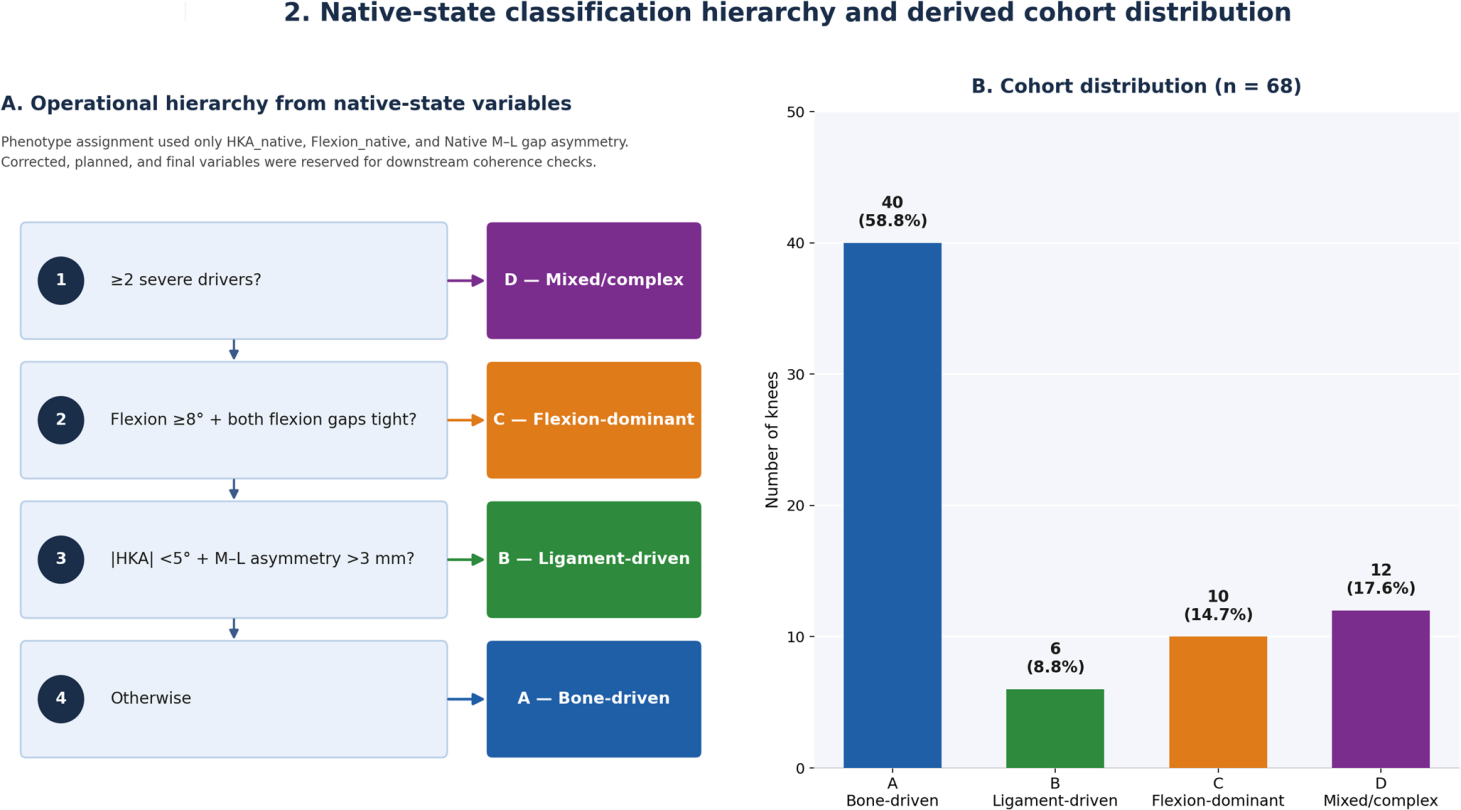
Preoperative classification hierarchy and derived cohort distribution. The hierarchy is applied in the order mixed/complex, flexion-dominant, ligament-driven, and bone-driven. Phenotype assignment is performed before corrected, planned, or final-state interpretation. The derived distribution was bone-driven 40/68 (58.8%), ligament-driven 6/68 (8.8%), flexion-dominant 10/68 (14.7%), and mixed/complex 12/68 (17.6%)

The full decision hierarchy is summarized in Fig. 2. A severe driver was defined as any of the following: absolute native HKA ≥ 10°, native flexion deformity ≥ 10°, or medial-lateral gap asymmetry > 5 mm in either extension or flexion. The 10° coronal threshold was anchored to the conventional mild/severe varus boundary used in prior TKA gap-balancing literature, in which an HKA varus angle ≥ 10° is associated with significantly larger flexion and extension gap differences than lesser deformity [13]; the same 10° magnitude was extended by symmetry to the sagittal plane (flexion deformity) given the comparable order-of-magnitude impact of fixed flexion contracture on gap behavior. The 5 mm gap-asymmetry threshold and the 8° flexion-dominant and 3 mm ligament-driven thresholds were not derived from receiver-operating-characteristic analysis or an independent reference population; rather, they were chosen heuristically during the formative 10-case series to give clinically separable, auditable categories, and we acknowledge this as a limitation addressed further in the Discussion and Limitations. Mixed/complex knees were assigned first when two or more severe drivers were present. Flexion-dominant knees were then defined by a native flexion deformity ≥ 8° in the presence of tight medial and lateral flexion gaps. Ligament-driven knees were defined by near-neutral coronal alignment (|HKA| <5°) and medial-lateral asymmetry > 3 mm. All remaining knees were classified as bone-driven. Corrected, planned, and final variables were not permitted to influence phenotype assignment.

### Internal coherence endpoints

Downstream endpoints were used as measures of internal coherence and were not involved in phenotype assignment. These endpoints included release escalation, corrected-state residual HKA, absolute plan-to-final HKA error, the proportion of cases within ± 2° of the planned HKA, strict mechanical balance, and physiological functional-alignment balance. Strict mechanical balance was defined as medial-lateral asymmetry ≤ 1 mm in both extension and flexion. The term “physiological” functional-alignment balance follows the convention used in prior gap-laxity and gap-balancing studies of the native, non-arthritic knee [8, 9], and was operationally defined in the present study by final gap measurements within the predefined envelope: medial extension 0 to + 2 mm, lateral extension 0 to + 3 mm, medial flexion − 1 to + 2 mm, and lateral flexion 0 to + 4 mm.

### Handling of reclassification and missing data

The operational hierarchy was reapplied to the locked dataset before analysis. Two cases originally labelled as ligament-driven in the working spreadsheet were reassigned by the objective hierarchy, with one reclassified to flexion-dominant and one to mixed/complex. No numeric intraoperative values were modified. Reduced denominators for plan-to-final HKA fidelity and final gap balance analyses reflected unavailable post-planning or final screen captures; unavailable endpoints were not imputed.

### Statistical analysis

Continuous variables are reported as mean ± standard deviation and, where appropriate, median and interquartile range. Proportions are reported with Wilson 95% confidence intervals. Because this was a single-cohort derivation and internal-coherence study, between-phenotype comparisons were considered exploratory and hypothesis-generating rather than confirmatory. Accordingly, the manuscript intentionally avoids language implying external validation of the proposed classification.

Inter-observer reliability of the classification was assessed in a separate blinded exercise. Five attending knee arthroplasty surgeons from the operating team, none of whom had access to the reference assignment, independently classified all 68 cases into the four phenotypes using only the preoperative variables (HKA, flexion deformity, and medial and lateral extension and flexion gaps) and the operational hierarchy described above. Cases were presented in randomized order under blinded identifiers. Agreement among the five raters was quantified using Fleiss’ kappa for overall multi-rater agreement and mean pairwise Cohen’s kappa, with overall percent agreement and full unanimity (identical classification by all five raters) also reported; a nonparametric bootstrap (2000 resamples over cases) was used to estimate the 95% confidence interval for Fleiss’ kappa. Kappa values were interpreted according to the Landis and Koch benchmarks.

## Results

### Cohort structure

The cohort included 68 consecutive primary robotic TKAs, comprising 51 varus knees (75.0%), 16 valgus knees (23.5%), and 1 neutral knee (1.5%). Mean age was 73.0 ± 7.7 years among the 67 cases with documented age, and 50 of 68 patients were women (73.5%). The implant design was CS in 60 cases, CR in 4, and PS in 4 (Table 1).

**Table 1:** Cohort profile and derived phenotype characteristics

Panel A. Cohort structure and preoperative profile (*N* = 68).							
Domain / variable			Result		Interpretation		
Cases, n			68		51 varus; 16 valgus; 1 neutral		
Age, years			73.0 ± 7.7		*n* = 67 documented		
Female sex, n (%)			50/68 (73.5%)		—		
Implant design			CS 60; CR 4; PS 4		Triathlon family		
Native HKA, °			4.3 ± 6.3		Positive = varus; negative = valgus		
|Native HKA|, °			6.7 ± 3.5		Native coronal burden		
Native flexion deformity, °			6.7 ± 6.8		Positive = flexion contracture		
Pre-cut deep MCL release			51/68		All varus knees		
Release escalation			1/68 (1.5%)		Moderate release in one varus case		
Posterior capsulotomy			0/68		None recorded		

Panel B. Derived phenotype distribution and preoperative signatures.							
Phenotype	*n* (%)	Deformity mix	|Native HKA|, °	Native flexion, °	ΔML extension, mm	ΔML flexion, mm	Severe‑driver count
A — Bone‑driven	40 (58.8%)	Varus 29, Valgus 10, Neutral 1	6.5 ± 3.0	5.0 ± 5.0	−2.6 ± 3.2	−3.3 ± 3.2	0.55 ± 0.50
B — Ligament‑driven	6 (8.8%)	Varus 6	3.1 ± 1.6	1.7 ± 5.7	−3.0 ± 1.3	−5.1 ± 2.7	0.33 ± 0.52
C — Flexion‑dominant	10 (14.7%)	Valgus 5, Varus 5	5.1 ± 2.9	14.6 ± 6.5	0.2 ± 2.9	−0.5 ± 0.9	1.00 ± 0.00
D — Mixed/complex	12 (17.6%)	Varus 11, Valgus 1	10.8 ± 3.0	8.3 ± 7.7	−4.2 ± 4.3	−4.7 ± 4.2	2.08 ± 0.29

HKA = hip-knee-ankle angle; CS = cruciate-substituting; CR = cruciate-retaining; PS = posterior-stabilized; MCL = medial collateral ligament. ΔML = medial minus lateral; negative values indicate relatively greater lateral laxity

### Formative pilot and final hierarchy-based assignment

The first 10 cases used for concept generation were retrospectively assigned to the proposed phenotypes and distributed as A = 5, B = 1, C = 1, and D = 3. After application of the finalized hierarchy to all 68 cases, the final phenotype distribution was A — bone-driven in 40/68 (58.8%), B — ligament-driven in 6/68 (8.8%), C — flexion-dominant in 10/68 (14.7%), and D — mixed/complex in 12/68 (17.6%). All 68 cases were classified using preoperative data alone (Table 1).

### Preoperative phenotype signatures

Bone-driven knees were the largest group and included most varus knees, a subset of valgus knees, and the single neutral knee. Ligament-driven knees were exclusively varus in this cohort and demonstrated low mean absolute HKA but clinically relevant medial-lateral asymmetry. Flexion-dominant knees included both varus and valgus cases and exhibited the greatest native flexion deformity. Mixed/complex knees were predominantly varus and demonstrated the highest burden of severe drivers as well as the largest corrected-state residual deformity (Table 1). The relationship between native coronal deformity, sagittal deformity, and gap asymmetry across the four phenotypes is mapped in Supplementary Figure S1. Native flexion deformity proved substantially correctable by final assessment across all phenotypes (overall native 6.7° ± 6.8° versus final 2.4° ± 1.6°). This correction was most pronounced in flexion-dominant knees (native 14.6° ± 6.5° versus final 2.9° ± 2.2°; mean correction 11.7° ± 6.9°) and least pronounced in ligament-driven knees, which had minimal native flexion deformity to begin with (native 1.7° ± 5.7°; mean correction − 1.0° ± 6.7°), indicating that flexion-dominant knees behaved as a largely correctable rather than fixed contracture in this cohort. Patellofemoral tracking and patellar-compartment ligament balance were not systematically captured in the robotic dataset and could not be analyzed by phenotype; this is addressed as a limitation.

### Release strategy and corrected-state behavior

The standardized release strategy was followed in all cases. Release escalation occurred in 1/68 cases (1.5%; 95% CI 0.3–7.9), and no posterior capsulotomy was recorded. Corrected-state residual deformity was greatest in mixed/complex knees, supporting the interpretation that this phenotype reflects a greater burden of severe coexisting deformity drivers rather than simply a larger coronal deformity.

### Plan-to-final fidelity and final balance

**Fig. 3:**
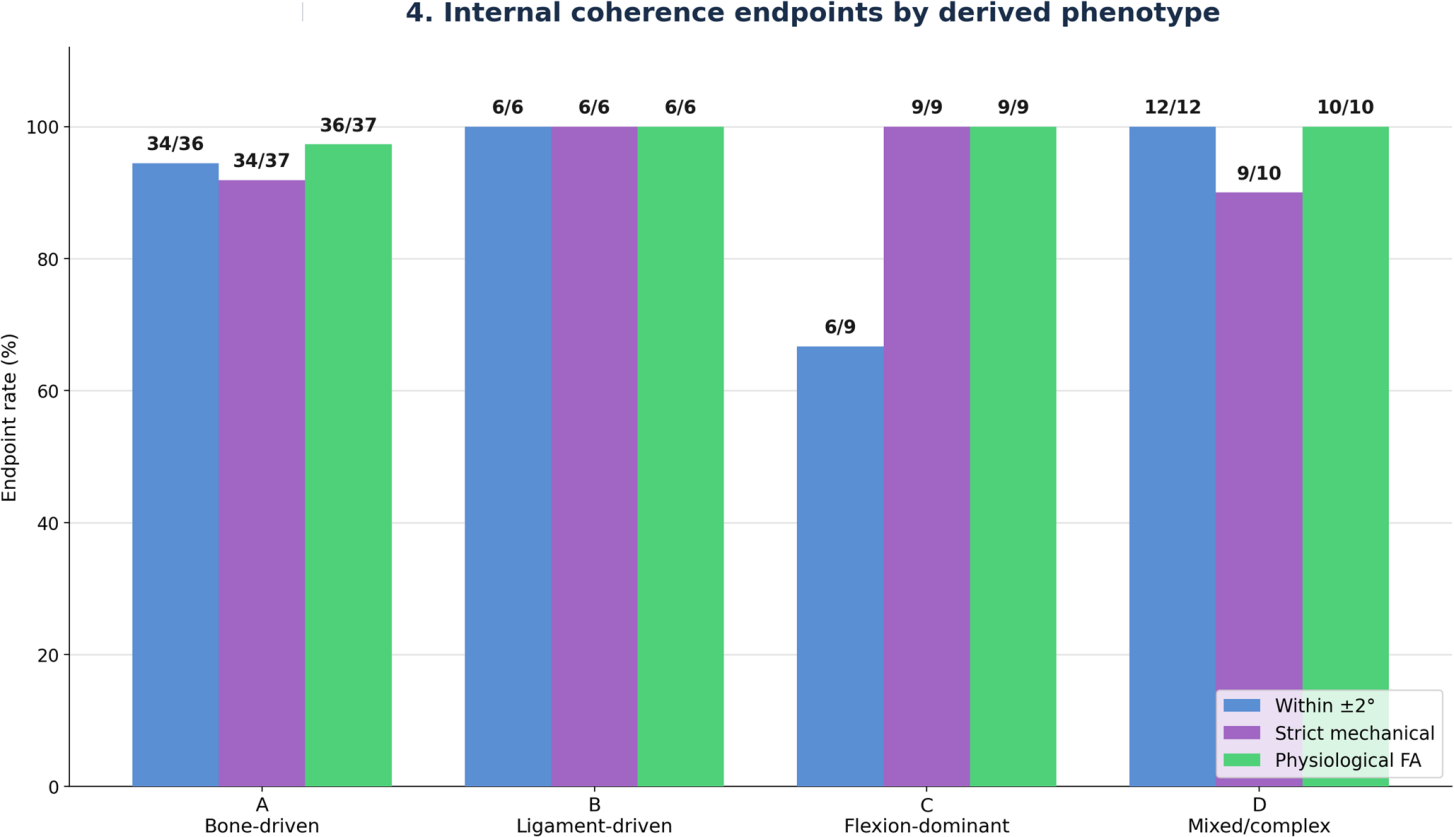
Internal coherence endpoints by phenotype. Endpoint denominators vary because not all post-planning and final screen captures contained complete data

Overall, 58/63 cases (92.1%; 95% CI 82.7–96.6) finished within ± 2° of the planned HKA. Strict mechanical balance was achieved in 58/62 cases (93.5%; 95% CI 84.6–97.5), and physiological functional-alignment balance was achieved in 61/62 cases (98.4%; 95% CI 91.4–99.7) (Table 2; Fig. 3). Flexion-dominant knees had the lowest within-±2° rate (6/9 (66.7%)), but achieved both strict mechanical and physiological balance in all evaluable cases. This pattern is consistent with a clinically plausible coronal–sagittal trade-off rather than a simple technical failure.

**Table 2:** Internal coherence and derivation roadmap

Panel A. Internal coherence endpoints by derived phenotype.							
Phenotype	*n*	|Corrected HKA|, °	Reducibility burden, °	|Plan error|, °	Within ± 2°	Strict mechanical balance	Physiological FA balance
A — Bone‑driven	40	0.94 ± 1.54	5.43 ± 2.31	0.88 ± 0.78	34/36 (94.4%)	34/37 (91.9%)	36/37 (97.3%)
B — Ligament‑driven	6	0.00 ± 0.00	3.08 ± 1.56	1.08 ± 0.58	6/6 (100.0%)	6/6 (100.0%)	6/6 (100.0%)
C — Flexion‑dominant	10	0.22 ± 0.51	4.39 ± 2.48	1.56 ± 1.13	6/9 (66.7%)	9/9 (100.0%)	9/9 (100.0%)
D — Mixed/complex	12	2.75 ± 1.67	8.00 ± 2.75	0.83 ± 0.65	12/12 (100.0%)	9/10 (90.0%)	10/10 (100.0%)

Panel B. Derivation, reclassification, and validation roadmap.							
Step		Dataset / source		Observed result		Interpretation	
Pilot formative series		First 10 consecutive cases		A = 5, B = 1, C = 1, D = 3		Concept generation and face-validity refinement only	
Locked derivation cohort		All 68 consecutive cases		A = 40, B = 6, C = 10, D = 12		Hierarchy reapplied to preoperative variables only	
Classification audit		Working labels vs. final hierarchy		2 labels changed; 0 numeric values altered		Protects methodologic reproducibility	
Internal coherence		Downstream endpoints		Release escalation 1/68; within ± 2° 58/63; FA balance 61/62		Supports plausibility but not external validation	
Next validation stage		Independent cohorts / reviewers		Inter-rater reliability, threshold sensitivity, PROM/radiograph correlation		Required before claiming universal taxonomy	

Reduced denominators reflect endpoint-specific unavailable screenshots. FA = functional alignment. This study derives and internally tests the framework; it does not constitute external validation

### Inter-observer reliability

Across the five independent surgeons, classification of the 68 cases showed almost perfect agreement. Overall percent agreement was 92.4%, and all five raters assigned an identical phenotype in 55 of 68 cases (80.9% full unanimity). The Fleiss’ kappa for multi-rater agreement was 0.88 (95% CI 0.81–0.93), and the mean pairwise Cohen’s kappa was 0.88 (range 0.86–0.91), both corresponding to almost perfect agreement on the Landis and Koch scale. Category-level agreement was highest for the bone-driven (A; 90.0% of rater pairs concordant) and ligament-driven (B; 88.2%) phenotypes, followed by mixed/complex (D; 83.9%), and was lowest for the flexion-dominant phenotype (C; 73.9%). The residual disagreement was almost entirely confined to borderline cases adjacent to the 8° sagittal and 5 mm asymmetry thresholds, most commonly between flexion-dominant and either bone-driven or mixed/complex assignments, consistent with the heuristic nature of these cutoffs (Supplementary Figure S2).

### Classification audit

Two working-sheet phenotype labels changed after the objective hierarchy was reapplied. Case 67 was reclassified from ligament-driven to flexion-dominant because native flexion deformity was 10° and both native flexion gaps were tight. Case 68 was reclassified from ligament-driven to mixed/complex because both severe coronal and ligament drivers were present. These changes affected only the phenotype assignment; no numeric data were modified (Table 2).

### Sample-size rationale and role of the formative 10-case series

The first 10 cases were used only as a formative concept-generation series. They were deliberately not treated as a validation sample and were not used to estimate definitive phenotype prevalence. Their role was to expose recurring preoperative patterns, refine the clinical language of the four drivers, and establish face validity before applying a locked hierarchical rule to the complete 68-case cohort.

This distinction is statistically important. With 10 cases, the probability of observing at least one example of a phenotype with true prevalence p is 1 − (1 − p)¹⁰. Thus, a 10-case pilot is highly likely to reveal very common patterns, but it is underpowered to reliably detect uncommon phenotypes or to estimate phenotype proportions with precision [14, 15]. For example, if a phenotype truly occurs in 20% of cases, a 10-case pilot has an 89.3% chance of observing it at least once; if the true prevalence is 10%, that probability falls to 65.1%, and if prevalence is 5%, it falls to 40.1% (Supplementary Table S1).

The minimum sample required for at least a 95% probability of observing one or more such cases is n ≥ ln(0.05)/ln(1 − p). The 10-case pilot is therefore defensible for framework generation and face-validity refinement, whereas the full 68-case cohort is the appropriate dataset for internal coherence testing and precision-based reporting.

For the actual full-cohort phenotype distribution observed in this study, Wilson confidence intervals remained appropriately reported because some phenotypes were uncommon: A, 40/68 (58.8%; 95% CI 47.0%-69.7%); B, 6/68 (8.8%; 95% CI 4.1%-17.9%); C, 10/68 (14.7%; 95% CI 8.2%-25.0%); and D, 12/68 (17.6%; 95% CI 10.4%-28.4%). These intervals reinforce that the present study should be interpreted as derivation plus internal coherence testing, not external validation.

## Discussion

The principal finding of this study is that preoperative alignment, flexion deformity, and medial-lateral gap asymmetry can be organized into four clinically interpretable driver phenotypes. This framework is neither a purely morphological classification nor a black-box clustering output. Rather, it represents a pragmatic approach derived from recurring intraoperative patterns and translated into an auditable hierarchy that can be applied at the robotic console before definitive planning.

This revision is conceptually important. The initial version of the framework risked implying that the classification had been imposed on the cohort. The present approach makes the developmental pathway explicit: a 10-case formative series was used to generate the concept and refine face validity, after which the hierarchy was operationalized and applied to all 68 cases using preoperative variables alone. Internal coherence was subsequently assessed using downstream workflow endpoints. This structure more accurately reflects how the classification was developed and tested.

The proposed classification differs from existing morphology-based systems. CPAK and related frameworks [6] describe the limb and joint-line morphology but do not directly identify whether the primary intraoperative driver is osseous, ligamentous, sagittal, or mixed. A driver-based phenotype seeks to define the problem that the surgeon must address before component-position optimization begins. The two approaches should therefore be viewed as complementary rather than competing frameworks.

This framework was derived exclusively on a CT-based, semi-active robotic-arm platform (MAKO), and its generalizability to other computer-assisted systems should not be assumed. Available systems differ substantially in their planning workflow, dynamic gap-acquisition method, and execution mechanism: MAKO and TSolution One require preoperative CT-based planning, whereas ROSA, CORI, and OMNIBotics rely on imageless or plain-radiograph-based intraoperative mapping; MAKO captures gap data only in full extension and at 90° of flexion via a single-radius femoral model, while other platforms acquire dynamic data across a continuous range of motion using varied stress-application methods [16]. Because the present preoperative thresholds were calibrated to MAKO’s specific gap-capture protocol, the same coronal, sagittal, and gap-asymmetry cutoffs may require platform-specific recalibration before being applied to ROSA, CORI, VELYS, or OMNIBotics data. We similarly did not stratify phenotype behavior by femoral implant design (cruciate-retaining, cruciate-substituting, or posterior-stabilized); because flexion-gap mechanics and posterior cruciate ligament tensioning differ materially between these designs, and our cohort was dominated by cruciate-substituting implants (60/68 cases), the interaction between implant constraint type and driver phenotype could not be adequately tested here and represents an important direction for future multi-platform, multi-implant validation. Finally, although this study did not compare functionally aligned robotic TKA against conventional jig-based instrumentation, robotic-assisted TKA as a category has been associated with significantly fewer mechanical alignment outliers and less deviation from neutral mechanical axis than conventional TKA in recent meta-analyses of randomized trials [3], providing external context for why precise preoperative characterization at the robotic console is clinically meaningful.

The flexion-dominant phenotype illustrates the potential value of this logic. These knees had the lowest within-±2° rate, yet achieved strict and physiological balance in all evaluable cases. Viewed through a narrow plan-to-final coronal lens, this finding could be interpreted as inferior execution. From a functional-alignment perspective, however, it may instead reflect a deliberate coronal–sagittal trade-off undertaken to restore the flexion-extension envelope. This observation is clinically relevant because the goals of robotic TKA extend beyond coronal alignment reproduction alone.

Mixed/complex knees illustrate a different concept. They had the highest burden of severe drivers and the greatest corrected-state residual deformity, yet maintained high plan-to-final fidelity and physiological balance. These findings support the value of sequential robotic assessment, in which the Preoperative state identifies the initial driver profile, the Corrected state quantifies residual deformity following the standardized soft-tissue protocol, the Planned state reflects component-mediated modulation, and the Final state verifies the achieved balance envelope.

The low release-escalation rate also supports the plausibility of the proposed workflow. Only one case required escalation beyond the default strategy, and no posterior capsulotomy was recorded. This does not prove that releases are unnecessary, but it suggests that functional alignment combined with robotic measurement can often address imbalance through measured component-position and resection modulation rather than extensive soft-tissue release [17, 18].

Beyond its immediate surgical workflow implications, the structured, quantifiable nature of this driver-based framework also speaks to a broader trajectory in robotic surgery. Because each phenotype is defined by reproducible, machine-captured variables rather than subjective intraoperative judgment, this type of framework is, in principle, well suited to downstream integration with artificial-intelligence-augmented decision-support and telementoring platforms, in which a remote or supervising surgeon could review the same preoperative driver signature in real time. Recent correspondence on AI-augmented autonomous telesurgery has emphasized that any such system must preserve surgeon explainability and final decision-making authority while clearly communicating the rationale behind each suggested maneuver [19]; an auditable, rule-based phenotype hierarchy of the kind proposed here—rather than an opaque machine-learning classifier—may be particularly well aligned with that requirement. Likewise, as robotic platforms increasingly explore remote and telementored configurations, a standardized, quantitative description of the native deformity driver could in the future help structure communication between an on-site surgeon and a remote mentor or supervising team, although this potential application remains speculative and was not tested in the present cohort [20].

The framework should be viewed as a structured research tool rather than a universal taxonomy. Future studies should evaluate inter-rater reliability using independent reviewers, assess threshold sensitivity, and determine whether phenotype assignment correlates with postoperative weight-bearing radiographs, PROMs, stiffness, instability, manipulation under anesthesia, and revision risk. Multicenter datasets would also permit supervised and unsupervised validation of the proposed thresholds.

### Limitations

This study has limitations. It is a single-surgeon, single-center derivation cohort using one robotic platform and one implant family. The derivation and coherence testing were performed in the same cohort, so external validation was not performed. Thresholds were selected for clinical interpretability and auditability rather than optimized through receiver-operating analysis or unsupervised clustering. Distraction force was standardized within the workflow but not independently recorded in Newtons. Several downstream endpoints had reduced denominators because not all post-planning and final screenshots contained complete values. Postoperative weight-bearing radiographs and patient-reported outcomes were not used in this analysis. Inter-observer reliability was assessed within the operating team and was almost perfect (Fleiss’ kappa 0.88); however, all raters belonged to a single center and applied the same operational rule, so intra-observer reproducibility over time and inter-observer reliability across independent institutions and differing levels of experience remain to be established. Specifically, the 10-case formative series should be interpreted as a concept-generation and face-validity sample only; probability-based sensitivity analysis shows that it cannot reliably capture rare phenotypes or provide precise prevalence estimates. Several of these limitations warrant particular emphasis. First, the numerical thresholds that define each phenotype (the 10° severe-driver boundary, the 8° flexion-dominant cutoff, the |HKA| <5° near-neutral boundary, and the 3 mm and 5 mm gap-asymmetry cutoffs) are, with the exception of the 10° coronal boundary anchored to prior gap-balancing literature [13], pragmatic and ultimately arbitrary choices made during the formative series; small changes in any of these cutoffs would reassign borderline cases between phenotypes, and a formal threshold-sensitivity analysis across a plausible range of values is required before the boundaries can be considered robust. Second, and importantly, the phenotype assignments in this study were generated and analyzed retrospectively from prospectively recorded robotic data; they were not used prospectively to guide any intraoperative decision, soft-tissue release, or component-position choice. The framework is therefore presented as a descriptive and hypothesis-generating tool, and the present data cannot support any claim that phenotype-guided decision-making improves outcomes. Third, MAKO acquires gap data only in full extension and at 90° of flexion and assumes a single-radius femoral component, so mid-flexion behavior was not characterized and the gap-based thresholds may not transfer to platforms or implant designs that model the flexion arc differently. Finally, while patient-reported outcome measures (WOMAC and Forgotten Joint Score) are being collected prospectively in this cohort, follow-up is presently incomplete and adherence varies across time points; we therefore deliberately did not perform a phenotype-stratified outcome analysis here, as doing so on partial data would risk precisely the kind of overinterpretation this framework is intended to avoid. Correlation of phenotype with mature clinical and radiographic outcomes is the central objective of planned follow-up work.

## Conclusion

In a consecutive 68-case cohort of functionally aligned robotic TKA, preoperative HKA, flexion deformity, and medial-lateral gap asymmetry revealed recurring driver patterns organized into bone-driven, ligament-driven, flexion-dominant, and mixed/complex phenotypes using preoperative variables alone. The framework demonstrated internal coherence across release escalation, corrected-state behavior, plan-to-final fidelity, and final balance, and showed almost perfect inter-observer reliability among five independent surgeons (Fleiss’ kappa 0.88), but should be interpreted strictly as a pragmatic, auditable derivation framework, not a validated taxonomy. External, multicenter validation and threshold refinement are required before it can inform clinical decision-making.

## Supplementary Information

Below is the link to the electronic supplementary material.

Figure S1 (Supplementary). Preoperative map of the 68-case cohort. Points are colored by derived phenotype. Point size reflects total native medial-lateral asymmetry in extension and flexion. Dashed lines show severe-driver thresholds for coronal and sagittal deformity.

Figure S2 (Supplementary). Inter-observer reliability of the driver-based phenotype classification. Five attending knee arthroplasty surgeons independently classified all 68 cases from preoperative variables under blinded conditions. Panel A shows the pairwise Cohen’s kappa between surgeons; Panel B shows the proportion of concordant rater pairs for each phenotype, with overall agreement, full unanimity, and the Fleiss’ kappa with its bootstrap 95% confidence interval. Agreement was almost perfect overall and lowest for the flexion-dominant phenotype.

## Data Availability

No datasets were generated or analysed during the current study.

## Declarations

### Use of AI

The authors used a large language model solely for AI-assisted copy editing (language, grammar, and formatting). No generative AI tool was used for study design, data analysis, interpretation, or the generation of scientific content. The authors reviewed and take full responsibility for the final manuscript.
