# Supplementary figures and images for: Preoperative gap phenotypes in functionally aligned robotic total knee arthroplasty: derivation and internal coherence of a driver-based classification
Source: J Robot Surg. 2026 Jul 20;20(1):703. doi: 10.1007/s11701-026-03655-4 (PMC13385008; doi:10.1007/s11701-026-03655-4)

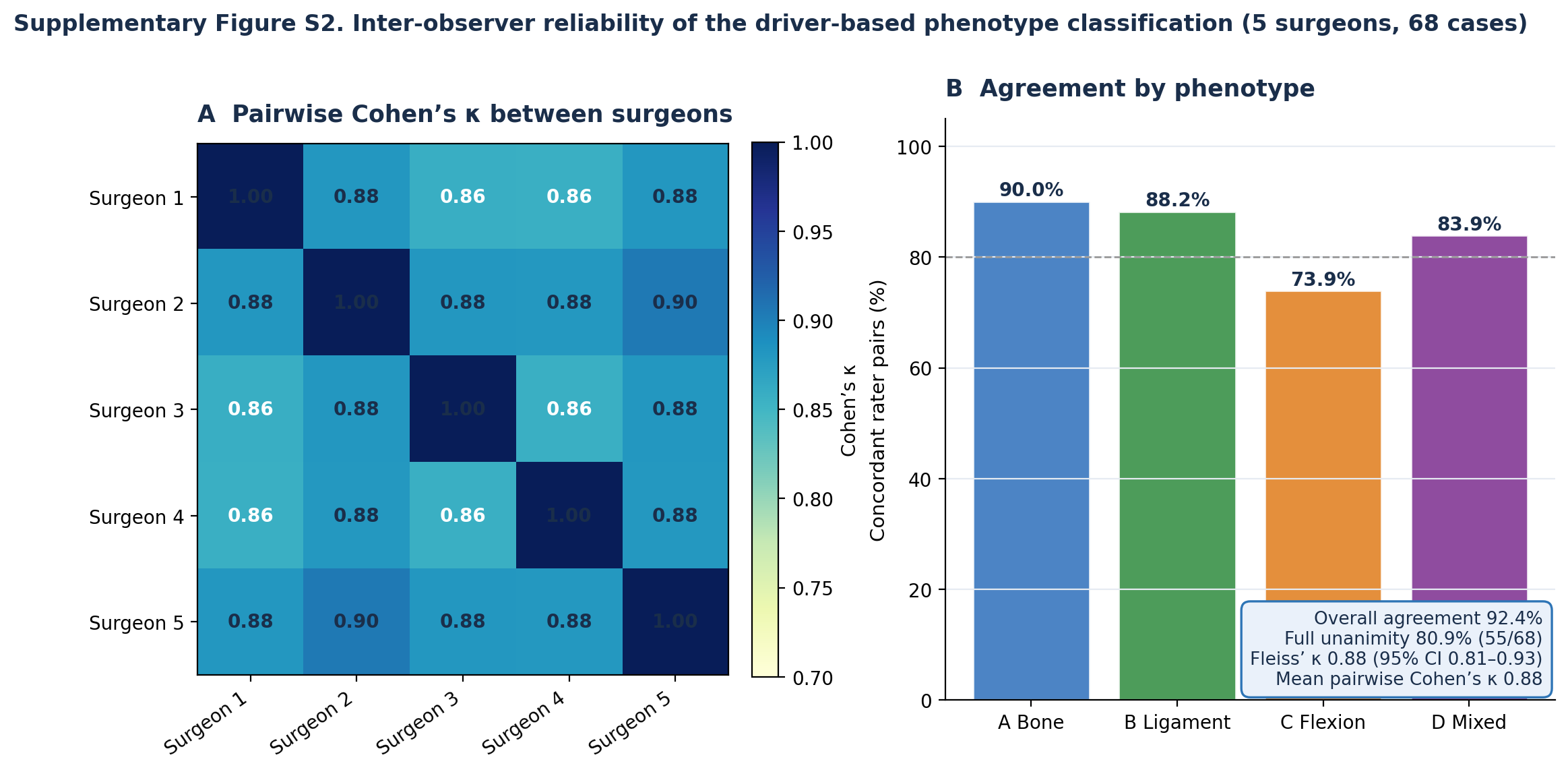

Supplement: Supplementary file 1 — Figure S1 (Supplementary). Preoperative map of the 68-case cohort. Points are colored by derived phenotype. Point size reflects total native medial-lateral asymmetry in extension and flexion. Dashed lines show severe-driver thresholds for coronal and sagittal deformity. [file 11701_2026_3655_MOESM1_ESM.png]

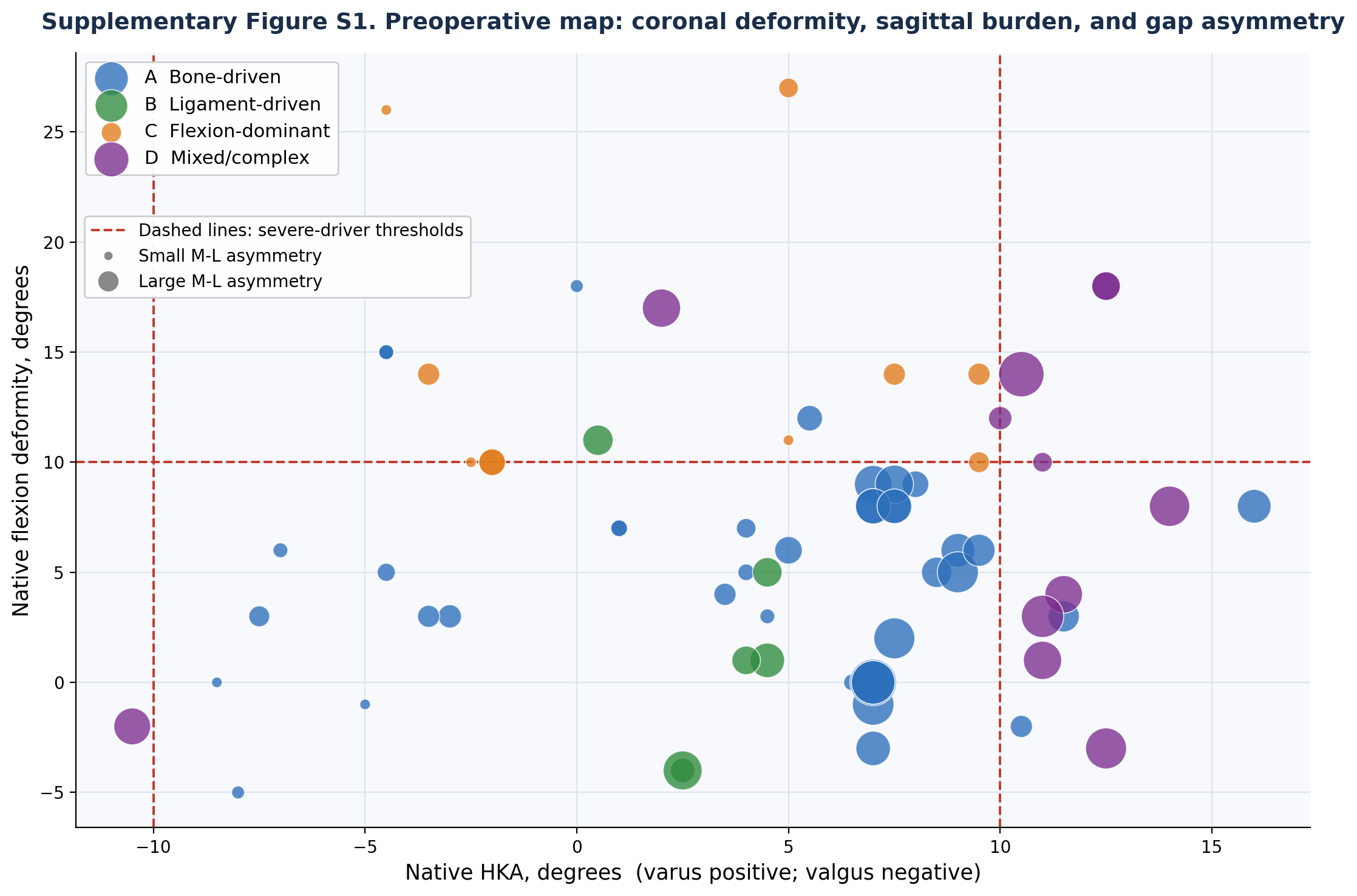

Supplement: Supplementary file 2 — Figure S2 (Supplementary). Inter-observer reliability of the driver-based phenotype classification. Five attending knee arthroplasty surgeons independently classified all 68 cases from preoperative variables under blinded conditions. Panel A shows the pairwise Cohen’s kappa between surgeons; Panel B shows the proportion of concordant rater pairs for each phenotype, with overall agreement, full unanimity, and the Fleiss’ kappa with its bootstrap 95% confidence interval. Agreement was almost perfect overall and lowest for the flexion-dominant phenotype. [file 11701_2026_3655_MOESM2_ESM.png]
